# Dystrophin Deficiency Causes Progressive Depletion of Cardiovascular Progenitor Cells in the Heart

**DOI:** 10.3390/ijms22095025

**Published:** 2021-05-10

**Authors:** Sarka Jelinkova, Yvonne Sleiman, Petr Fojtík, Franck Aimond, Amanda Finan, Gerald Hugon, Valerie Scheuermann, Deborah Beckerová, Olivier Cazorla, Marie Vincenti, Pascal Amedro, Sylvain Richard, Josef Jaros, Petr Dvorak, Alain Lacampagne, Gilles Carnac, Vladimir Rotrekl, Albano C. Meli

**Affiliations:** 1 Department of Biology, Faculty of Medicine, Masaryk University, Kamenice 5/A3, 62500 Brno, Czech Republic; sarka.jelinkova89@gmail.com (S.J.); petrfoj@hotmail.com (P.F.); deborah.beckerova@gmail.com (D.B.); pdvorak@med.muni.cz (P.D.); 2 ICRC, St Anne’s University Hospital, Pekařská 53, 65691 Brno, Czech Republic; jaros@med.muni.cz; 3 PhyMedExp, University of Montpellier, INSERM, CNRS, 34295 Montpellier, France; yvonne.sleiman@umontpellier.fr (Y.S.); franck.aimond@inserm.fr (F.A.); amandafinan@yahoo.com (A.F.); gerald.hugon@inserm.fr (G.H.); valerie.scheuermann@inserm.fr (V.S.); olivier.cazorla@inserm.fr (O.C.); m-vincenti@chu-montpellier.fr (M.V.); p-amedro@chu-montpellier.fr (P.A.); sylvain.richard@inserm.fr (S.R.); alain.lacampagne@inserm.fr (A.L.); gilles.carnac@inserm.fr (G.C.); 4 Pediatric and Adult Congenital Cardiology Department, M3C Regional Reference CHD Center, CHU Montpellier, 371 Avenue du Doyen Giraud, 34295 Montpellier, France; 5 Department of Histology and Embryology, Faculty of Medicine, Masaryk University, Kamenice 5/A1, 62500 Brno, Czech Republic

**Keywords:** duchenne muscular dystrophy, *mdx* mouse, cardiovascular progenitors, c-kit, genomic instability, dilated cardiomyopathy

## Abstract

Duchenne muscular dystrophy (DMD) is a devastating condition shortening the lifespan of young men. DMD patients suffer from age-related dilated cardiomyopathy (DCM) that leads to heart failure. Several molecular mechanisms leading to cardiomyocyte death in DMD have been described. However, the pathological progression of DMD-associated DCM remains unclear. In skeletal muscle, a dramatic decrease in stem cells, so-called satellite cells, has been shown in DMD patients. Whether similar dysfunction occurs with cardiac muscle cardiovascular progenitor cells (CVPCs) in DMD remains to be explored. We hypothesized that the number of CVPCs decreases in the dystrophin-deficient heart with age and disease state, contributing to DCM progression. We used the dystrophin-deficient mouse model (*mdx)* to investigate age-dependent CVPC properties. Using quantitative PCR, flow cytometry, speckle tracking echocardiography, and immunofluorescence, we revealed that young *mdx* mice exhibit elevated CVPCs. We observed a rapid age-related CVPC depletion, coinciding with the progressive onset of cardiac dysfunction. Moreover, *mdx* CVPCs displayed increased DNA damage, suggesting impaired cardiac muscle homeostasis. Overall, our results identify the early recruitment of CVPCs in dystrophic hearts and their fast depletion with ageing. This latter depletion may participate in the fibrosis development and the acceleration onset of the cardiomyopathy.

## 1. Introduction

Duchenne muscular dystrophy (DMD, prevalence 1/5000) is an X-linked genetic disorder causing the wasting of skeletal muscles, resulting in severe disability and premature death in young men [1]. In addition to skeletal muscle loss of function, cardiac muscle is nearly always affected in DMD [2] with the development of a dilated cardiomyopathy (DCM) starting by the end of the second decade of the DMD patient’s life [3,4]. As DMD patient care has improved over the years, heart failure has become the prevalent cause of mortality worldwide in DMD patients [5,6] and also in patients suffering from the less severe Becker muscular dystrophy (BMD). Both forms of the disease are related to dystrophin gene mutations.

The dystrophin deficiency in cardiomyocytes eventually leads to contractile failure followed by death [7]. The molecular and cellular mechanisms causing myocyte death are unclear and have been mostly investigated for skeletal muscle [8]. These reports have suggested impairment of calcium (Ca^2+^) homeostasis and other ion fluxes [9,10,11] either due to a compromised plasma membrane integrity, deregulation of stretch-activated channels, or stretch-activated-channel-induced release of reactive oxygen species (ROS) that can trigger a sarcoplasmic reticulum (SR) Ca^2+^ leak [10]. Dystrophin deficiency also causes a leaky ryanodine receptor/Ca^2+^ release channel (RyR1) associated with early post-translational modifications including S-nitrosylation and calstabin1 depletion in *mdx* skeletal muscle [12]. Abnormal intracellular Ca^2+^ handling increases the influx of Ca^2+^ that triggers necrosis and cell death in DMD. We and others have described similar defects in the heart with abnormal Ca^2+^ homeostasis and ryanodine receptor (RyR2) dysfunction [12,13]. The deregulation of other ion complexes such as the voltage gated channel Nav1.5 in dystrophin-deficient cardiomyocytes has been reported [14].

In skeletal muscle, the population of satellite cell progenitors is affected, which has been implicated in the disease progression [15,16]. Dystrophinopathy results in myocyte death inducing muscle satellite cell activation [17] and increased differentiation [18,19] resulting in their eventual depletion [20]. Such a mechanism is unclear in the heart. Higher age-dependent fibrosis in *mdx* muscle and heart has been reported with early sarcolemmal damage induced by inflammation and macrophage infiltration [21,22]. Although DCM involves primarily cardiomyocyte damage, increasing attention has been given to non-muscular cell fractions, including cardiac fibroblasts [23,24,25] and endothelial cells [26,27,28,29], respectively involved in extracellular matrix and vascular remodeling. Other stromal cells possibly involved in cardiac homeostasis, survival, and disease dynamics are cardiovascular progenitor cells (CVPCs) [30], among which the CD117^+^ (also named c-kit)/CD45^−^ fraction is the main focus of recent studies [31,32,33]. We recently showed that CVPCs are activated and recruited to the site of cardiac injury [34], supporting the hypothesis of their paracrine regulation of cardiac function in pathophysiological conditions [31,35,36]. We found a CVPC depletion associated with late-stage pathological remodeling of cardiac muscle in BMD patients [37]. Nevertheless, the role and fate of CVPCs in the heart and cardiac pathology, particularly DMD-associated DCM, remains to be elucidated. Thus, in this study we investigated the chronological involvement of the CVPC population, in particular the c-kit^+^/CD45^−^ cells, in the progression of DMD-associated DCM in *mdx* dystrophic hearts.

## 2. Results

### 2.1. Mdx Cardiac Tissue Displays Decreasing Levels in C-Kit Receptor mRNA Expression with Ageing

**Figure 1:**
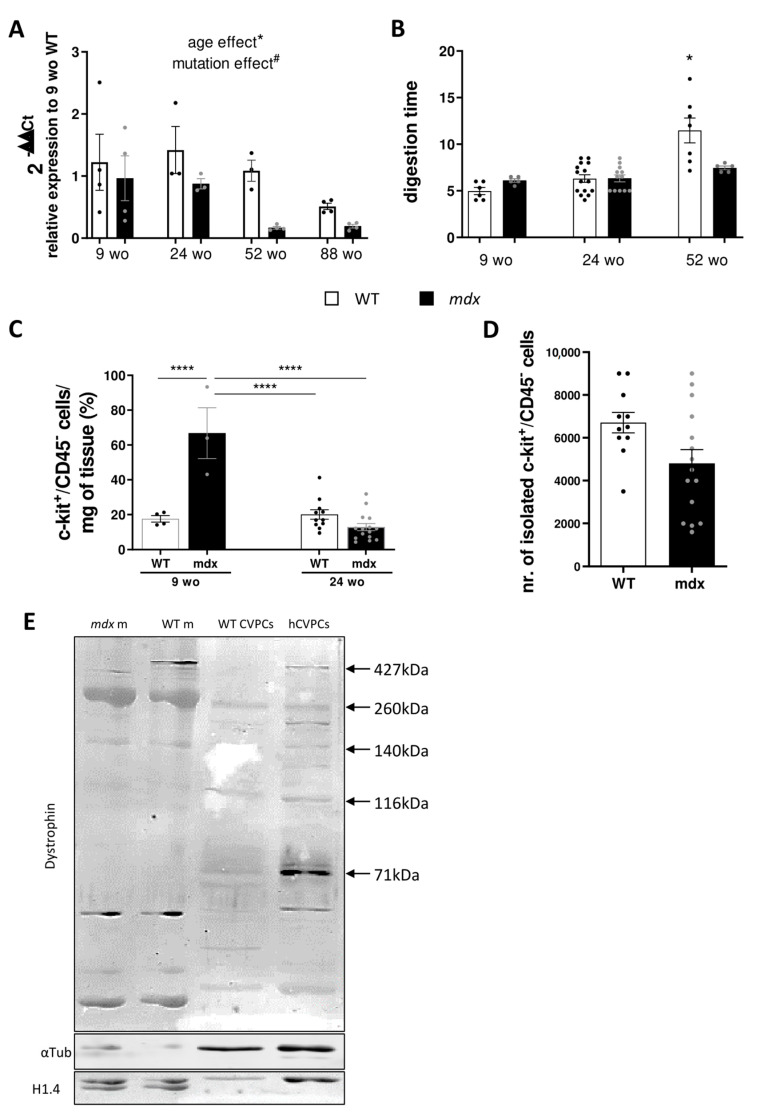
C-kit expression decreases in *mdx* animals in an age-dependent manner. (**A**) Bar graphs summarizing the relative *c-KIT* gene expression analyzed by RT-PCR in WT (open bars and black dots) and *mdx* (black bars and grey dots) cardiac left ventricles. Data are shown as mean ± SEM (*n* = 3–4 animals in each group). The presented value was calculated as 2^-ΔΔCt^ compared to housekeeping gene and 9 wo WT animals. Statistical difference was calculated by two-way ANOVA with Tukey’s post-hoc test with mutation (# *p* < 0.05 for WT compared to *mdx*) and age (* *p* < 0.05 for *mdx* ageing mouse) as evaluation criteria. (**B**) Bar graphs summarizing the digestion time (in minutes) to dissociate the mouse heart. Data are shown as mean ± SEM (*n* = 5–15 animals in each group). Kruskal–Wallis test with Dunn’s multiple comparison was used for statistical evaluation (* *p* < 0.05). (**C**) Ratio of c-kit^+^/CD45^−^ cells isolated by MACS after cardiomyocyte filtration from 9 wo and 24 wo WT (open bars and black dots, *n* = 4–11) and *mdx* (black bars and grey dots, *n* = 3–16) mice, normalized by heart weight. Data are shown as mean ± SEM. Statistical significance was calculated using two-way ANOVA with Tukey’s post-hoc test (**** *p* < 0.0001). (**D**) Number of c-kit^+^/CD45^−^ cells isolated by MACS after cardiomyocyte filtration in 24 wo WT mice (open bars and black dots, *n* = 11 animals) and 24 wo *mdx* mice (black bars and grey dots, *n* = 15 animals). (**E**) Illustrative images of Western blot (*n* = 3 repetitions) to evaluate the dystrophin expression in mouse (WT CVPCs) and human c-kit^+^ (hCVPCs) isolated cells. The WT mouse skeletal muscle tissue (WT m) was used as positive control, while *mdx* skeletal muscle tissue (*mdx* m) was used to show no expression of the long isoform of dystrophin (Dp427) in *mdx* animal. The α-tubulin and basic histone H1.4 were used as loading controls.

We first analyzed the relative gene expression of *c-KIT* gene in different age-matched groups of 9, 24, 52, and 88 week-old (wo) WT and *mdx* mice. Based on previous work, these ages span the full range of the cardiac symptoms from young adult without symptoms through to adult with onset of cardiac symptoms to fully developed cardiac symptoms in old animals with late-stage heart failure [13]. The relative gene expression of *c-KIT* was evaluated by RT-PCR. At 9 and 24 wo, we observed similar expression between WT and *mdx* mice. A severe decrease with ageing was observed in older *mdx* animals (*p* = 0.0151 for mutation factor, *p* = 0.0121 for ageing factor). Interestingly, WT animals presented change in *c-KIT* expression only at 88 wo of age and not at the previously investigated age (Figure 1A).

### 2.2. Mdx CVPCs Show Early In Vivo Amplification Followed by Depletion and Low Resilience in In Vitro Culture

We next isolated the c-kit^+^/CD45^−^ cells in control and *mdx* hearts by enzymatic digestion through the Langendorff perfused heart method followed by magnetic sorting of the cell suspension. As cardiac fibrotic deposit is known to occur in *mdx* hearts [38], we evaluated whether the fibrosis could affect the digestion efficiency by evaluating the full digestion time. No significant difference was seen with an exception for the oldest WT animals with prolonged digestion time caused by blood clot formation (Figure 1B). The quality, viability, number, and shape of the cardiomyocytes were checked under a microscope. The ratio of isolated c-kit^+^/CD45^−^ cells over mg of cardiac tissue was higher in 9 wo *mdx* mice compared with age-matched WT (17.6 ± 1.8% in WT vs. 66.8 ± 14.5% in *mdx*, *p* ≤ 0.0001, Figure 1C). There was no difference in 24 wo control and *mdx* mice (20.1 ± 2.7% in WT vs. 12.8 ± 2.0% in *mdx*, Figure 1C). Using MACS, we estimated lower isolated cell numbers in adult *mdx* samples (4800 ± 2500 cells vs. 6700 ± 1600 cells from *mdx* and WT respectively, *p* = 0.0362, Figure 1D). These cells were cultivated in vitro for further investigation. However, the plating efficacy was much lower compared to WT, with only sparse cells attaching to the cultivation surfaces, and thus in vitro cultivation could not be established. Further experiments on the CVPCs were thus conducted immediately after isolation without prolonged in vitro cultivation.

### 2.3. Different Expressed Dystrophin Isoforms in Mouse and Human CVPCs

Next, we evaluated whether the CVPC population expresses dystrophin isoforms that could impair the function of CVPCs in *mdx* mice. Dystrophin expression was analyzed by Western blot. Skeletal muscle of WT and *mdx* mice was loaded as reference. As expected, we found no expression for the high molecular weight (Dp427) dystrophin isoform in the *mdx* muscle compared to the WT muscle (Figure 1E). The mouse WT CVPCs only express shorter isoforms including bands at 260, 140, 116, and 71 kDa but no Dp427. In contrast, the human control CVPCs have a different expression pattern of the dystrophin isoforms, including the high molecular weight (Dp427) and ubiquitous isoform Dp71 (Figure 1E).

### 2.4. Increased Resident CVPCs at Early Stage Followed by Premature Depletion in Dystrophin-Deficient Heart

**Figure 2:**
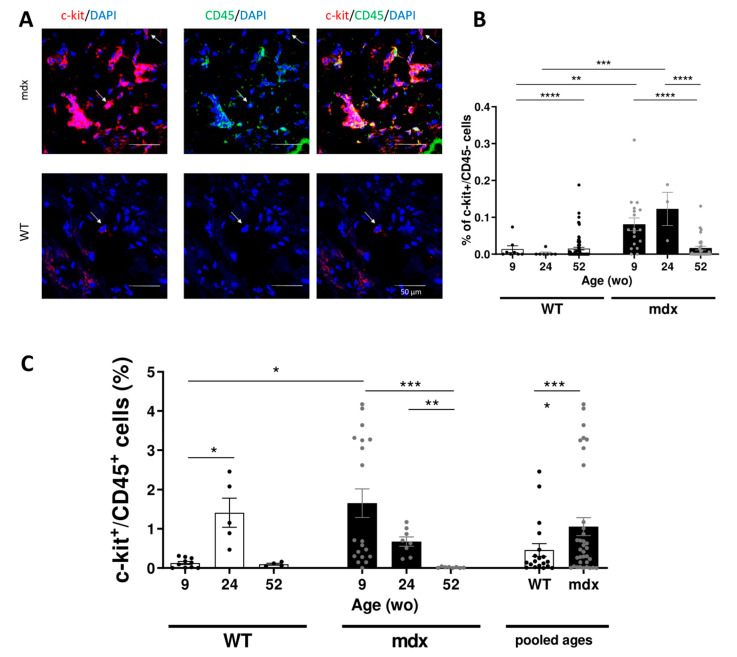
Recruitment of CVPCs in *mdx* hearts. (**A**) Representative histological analysis images of c-kit and CD45 immunolabeling. CVPCs were evaluated only as cells with positive signal for c-kit (red) and without signal for CD45 (green). Individual cells with these criteria are identified by arrows on the images. C-kit^+^/CD45^−^ cells were evaluated as mast cells. Scale bars: 50 μm. (**B**) Bar graphs showing the percentage of c-kit^+^/CD45^−^ cells relative to the total number of cells in WT (open bar and black dots) and *mdx* (black bar and grey dots). Data are shown as mean ± SEM (*n* = 3–20 sections per group; three animals were analyzed for the 9 wo and 52 wo groups, and two animals were analyzed for the 24 wo group). The statistical significance was calculated by two-way ANOVA and Tukey’s multiple comparison test (* *p* < 0.05, ** *p* < 0.01, *** *p* < 0.001). (**C**) Bar graphs showing the percentage of inflammatory mast cells (c-kit^+^/CD45^−^) at different ages in WT (white bars and black dots) and *mdx* (black bars and grey dots) hearts. These mast cells were evaluated from the labeled sections shown in Figure 2A. Data are shown as mean ± SEM. Statistical significance was calculated by Kruskal–Wallis test and Dunn’s multiple comparisons for each individual age group of WT and *mdx* hearts and by Student’s t-test for pooled age groups (* *p* < 0.05, ** *p* < 0.01, *** *p* < 0.001, **** *p* < 0.0001).

**Figure 3:**
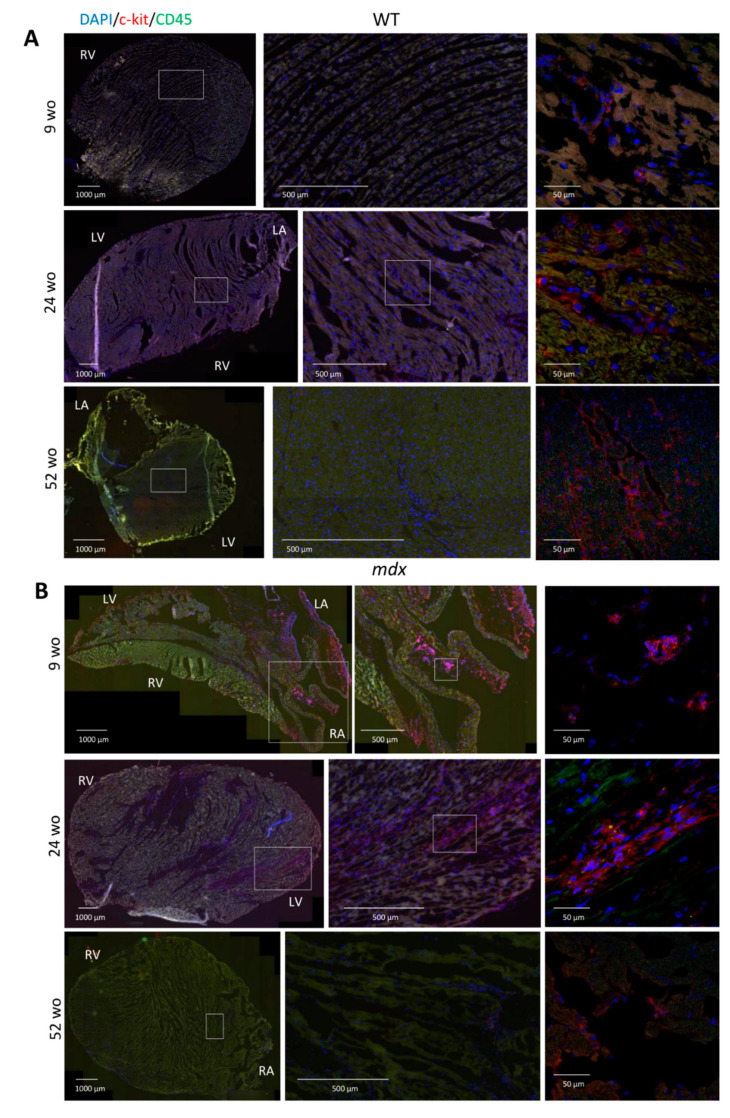
Abnormal age-dependent CVPC localization in the *mdx* heart. (**A**) Representative images of CVPC localization in WT hearts at the age of 9 (upper panels, *n* = 3 animals), 24 (middle panels, *n* = 2 animals), and 52 wo (lower panels, *n* = 4 animals). (**B**) Representative images of CVPC localization in *mdx* hearts at the age of 9 wo (upper panel, three animals), 24 wo (middle panel, two animals) and 52 wo (lower panel, four animals). RV: right ventricle, LV: left ventricle, RA: right atrium, LA: left atrium. The c-kit protein was labeled in red, CD45 protein in green, and the nuclear DNA was counterstained with DAPI in blue. The detailed CVPC image taken by confocal microscopy was supplemented to the overview of the slice taken on fluorescent microscope and exactly positioned in the figure relative to the overall slice (left panels taken by confocal microscope, right and middle panel show detailed section marked in white rectangle). For each image the scale bar is indicated.

To further explore the quantity of c-kit^+^/CD45^−^ in *mdx* mice along with ageing, immunofluorescence analysis was performed on cryosections of flash frozen heart. We determined the proportion of c-kit^+^/CD45^−^ cells relative to all cells identified by DAPI staining in the myocardium. We found a higher number of CVPCs in 9 wo *mdx* hearts (0.09 ± 0.07%) compared with WT hearts (0.01 ± 0.02%) (Figure 2A,B). This difference remained in older animals between *mdx* (0.12 ± 0.08%) and WT mice (0.003 ± 0.008%) at the age of 24 wo, corresponding to the onset of the cardiac fibrosis (Figure 2A,B). In WT hearts, only individual cells were randomly found localized throughout the myocardium as illustrated in Figure 3A. The localization of the CVPCs in *mdx* hearts was mostly in the right atrium and sporadically in the epicardial area in the 9 wo age group with re-localization into ventricular myocardium in 24 wo suggesting injury activated recruitment of the CVPCs as illustrated in Figure 3B. As CVPCs are thought to be involved in cardiac injury, we next evaluated whether inflammatory cell population is increased in the *mdx* heart. To that effect, we focused on the mast cells, known to be involved in the inflammatory process and specifically expressing the c-kit^+^/CD45^+^ markers [39]. We found that the *mdx* hearts exhibited a higher level of c-kit^+^/CD45^+^ mast cells at 9 wo. While the percentage of mast cells increased at 28 wo in the WT heart, no change was observed in the *mdx* heart at the same age (Figure 2C). However, *mdx* heart displayed deep depletion of the mast cells at 52 wo in contrast with those in WT heart. When ages were pooled, the *mdx* heart exhibited a higher percentage of mast cells compared to the WT heart (1.37 ± 1.30% in *mdx* to 0.08 ± 0.09% in WT; Figure 2C).

### 2.5. Early-Stage CVPC Recruitment Followed by Depletion Is Correlated with Fibrotic Development and Cardiac Dysfunction in Mdx Hearts

**Figure 4:**
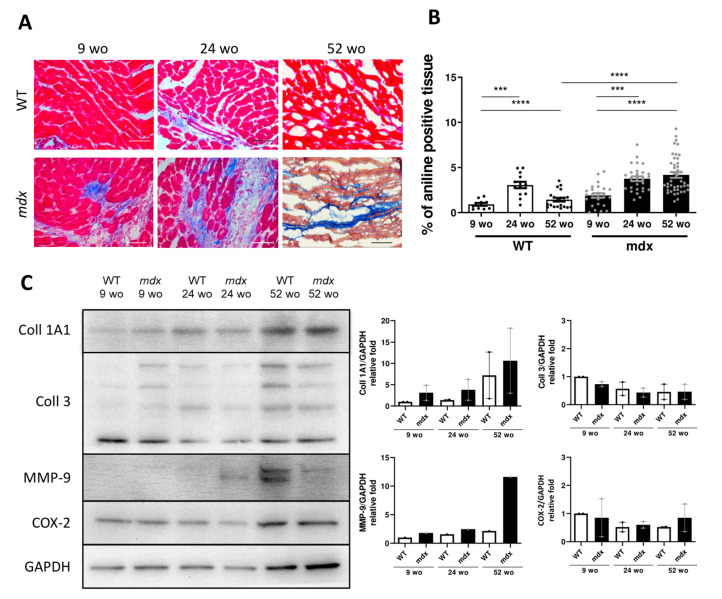
Fibrotic deposit and cardiac dysfunction correlate with decreasing CVPC presence in *mdx* heart. (**A**) Representative images of histological analysis stained using Masson trichrome technique showing myocytes (in red) and collagenous fibrotic tissue (in blue) in the left ventricle of WT and *mdx* hearts at 9, 24, and 52 wo. Line represents 100 µm. (**B**) The ratio of red and blue stained tissue was evaluated in WT hearts (open bars and black dots, *n* = 4–11 slices/3 animals per group) and *mdx* hearts (black bars and grey dots, *n* = 3–16 slices/3 animals per group) at the age of 24 wo and further at 52 wo. Statistical significance was calculated by Kruskal–Wallis test and Dunn‘s multiple comparison post-hoc test (*** *p* < 0.001, **** *p* < 0.0001). (**C**) Western blot analysis of collagen proteins and inflammatory proteins in the cardiac tissues. Left panel shows representative images of collagen 1A1 (Coll 1A1), collagen 3 (Coll 3), cyclooxygenase 2 (COX-2), and matrix metalloproteinase 9 (MMP-9) compared to the GAPDH control. The right panels show the normalized densitometry of each protein normalized by GAPDH content of WT (open bars, *n* = 2 animals) and *mdx* (black bars, *n* = 2 animals).

Next, we hypothesized that the progressive CVPC depletion in *mdx* is correlated with pathological cardiac remodeling known to be associated with fibrosis and left ventricular dysfunction. We first evaluated the progression of the cardiac remodeling with ageing by analyzing the collagen deposition as a main marker of fibrosis. We employed Masson’s trichrome staining on WT and *mdx* left ventricle of 9, 24, and 52 wo animals (Figure 4A,B). We observed similar collagen deposition in the *mdx* and WT hearts of 9 wo animals (0.8 ± 0.4% and 1.8 ± 1.1% of aniline blue positive tissue for WT and *mdx*, respectively). This level of collagen remained constant in WT animals with ageing (3.1 ± 1.3% in 24 wo and 1.4 ± 0.9% in 52 wo). However, the collagen content increased progressively in the *mdx* hearts with ageing (3.8 ± 1.3% at 24 wo and 4.2 ± 2% at 52 wo, Figure 4A,B). We next analyzed the composition of collagen isoforms, including collagen 1A1 and 3, by Western blotting (Figure 4C). We observed no statistical difference in the collagen 1A1 protein expression in the WT and *mdx* hearts, although a tendency was visible for a higher expression in the *mdx* (Figure 4C). We found no difference in the collagen 3 protein expression between WT and *mdx* hearts. We evaluated the protein expression level of the matrix metalloprotease MMP-9 as this protein has been suggested to have important roles in the skeletal muscle dystrophy progression [40]. However, no change was observed between WT and mdx hearts at any age, although the 52 wo heart displayed a tendency for higher MMP-9 expression compared to WT (Figure 4C). To further evaluate the inflammation level in the mdx hearts, we also assayed the cyclooxygenase-2 (COX-2) protein as it has been involved in DMD [41]. No change was observed at any age in WT and *mdx* hearts (Figure 4C).

**Figure 5:**
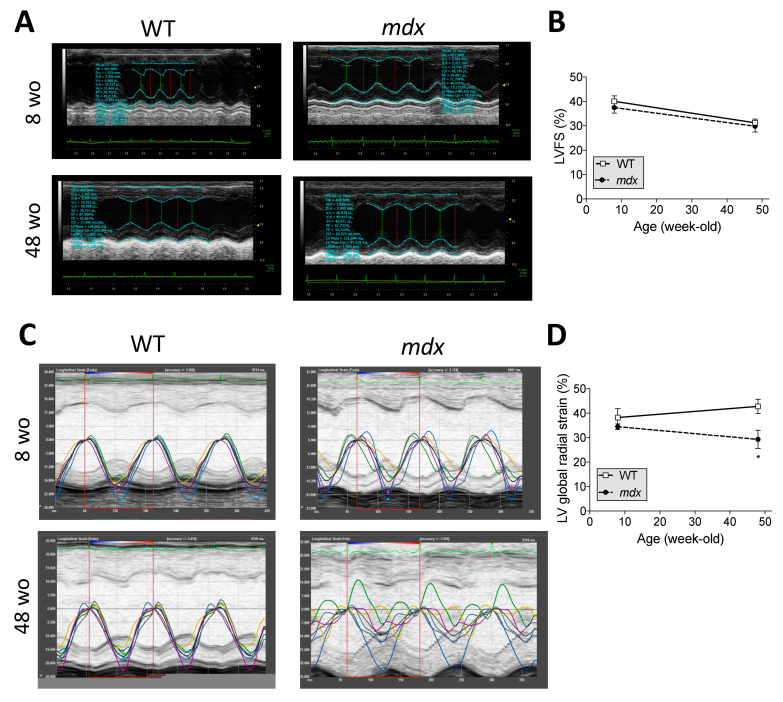
LV global strains are altered before LV ejection fraction in *mdx* mice. (**A**) M-mode images in WT and *mdx* at 8 and 48 wo. (**B**) Percentage (%) of the left ventricular fractional shortening (LVFS) in WT and *mdx* mice from 8 to 48 wo. (**C**) High-frequency ultrasound-based two-dimensional speckle-tracking (STE) images in WT and *mdx* at 8 and 48 wo. (**D**) Percentage (%) of the left ventricular (LV) global radial global strain in WT and *mdx* mice from 8 to 48 wo. Data are shown as mean ± SEM (*n* = 10 animals/group). A two-way ANOVA test was performed. * *p* < 0.05, *mdx* vs. WT mice at the same age.

We next evaluated the age-dependent cardiac dysfunction in WT and *mdx* mice using conventional M-mode and high-frequency ultrasound-based two-dimensional speckle-tracking echocardiography of the left ventricle. With the conventional echocardiography, no change was observed in the percentage of LV fractional shortening (LVFS), although there was a tendency to slow decrease in LVFS in both groups (Figure 5A,B). However, with the highly sensitive ultrasound technique, we found an age-dependent decreasing slope of the global radial strain in *mdx* mice when compared to WT. At 52 wo, a decrease in LV global strain was found (42.8 ± 2.9% vs. 29.3 ± 3.7% in WT and *mdx*, respectively) (Figure 5C,D).

### 2.6. DNA Damage and Oxidative Stress in CVPCs

**Figure 6:**
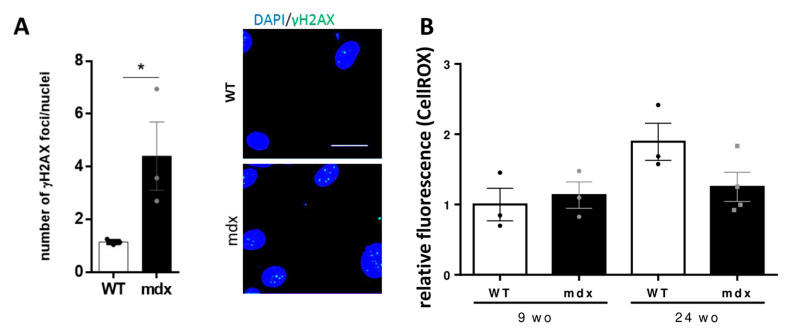
DNA damage is increased in *mdx* CVPCs. (**A**) Bar graphs showing the DNA double stranded breaks in isolated CVPCs from 24 wo WT and *mdx* mice as illustrated with the two images on the right. The DNA damage was evaluated as the ratio of γH2AX foci number (in green) per total number of nuclei (DAPI labeled, in blue) per image. At least 20 cells were evaluated from each animal; the average from each animal was used for statistics with three animals per group (* *p* < 0.05). The scale bar represents 20 µm. (**B**) Bar graphs showing the relative fluorescence of ROS production using CellROX dye on freshly isolated CVPCs of 9 and 24 wo mice. At least three animals per group were used. The values are represented in the bar graphs for WT (open bars and black circles/dots) and *mdx* (black bars and grey dots). The statistical difference was calculated using two-way ANOVA.

Recently, we showed that dystrophin deficiency causes oxidative stress and DNA double strand breaks in DMD human pluripotent stem cells [42]. Thus, we aimed to verify whether dystrophin deficiency leads to similar defects in the isolated CVPC population in *mdx* mice. Since we have shown drastic depletion of CVPCs in 52 wo animals, we hypothesized that the damage occurs before 52 wo. Therefore, we explored the DNA damage in the isolated CVPCs using immunocytological analysis in the 24 wo mice. Evaluation of the accumulated histone H2A phosphorylated on Ser192 (γH2AX) was performed, detecting double stranded breaks as foci in the nucleus (Figure 6A). The CVPCs from *mdx* animals showed a significant increase (4 times more) in DNA damage compared to CVPCs from age-matched WT animals. As DNA damage is associated with oxidative stress [42], we also evaluated the oxidative stress in the CVPCs. We observed no difference in oxidative stress, evaluated with the CellROX probe, between *mdx* and WT CVPCs (Figure 6B).

## 3. Discussion

In the present study, we showed, for the first time, that the dynamics of the CVPC population change with ageing in the *mdx* heart and correlate with the progression of the cardiac disease onset. Most of the studies related to DMD-associated DCM have focused on the pathological mechanisms related to dysfunction of the myocardium and morphological damage. Here, for the first time, we aimed at evaluating the impact of dystrophin deficiency on the capacity of the cardiac tissue to maintain its CVPC population and therefore its regenerative capacity. CVPCs were previously isolated from the heart of the golden retriever muscular dystrophy (GRMD) model of DMD [15]. They showed longer cell number doubling time in culture and lower telomere copy number, pointing to the limitations in expansion capabilities. A lower efficacy in cardiac differentiation was also found in GRMD-CVPCs compared to WT. Furthermore, it has been proven that CVPCs do not differentiate into cardiomyocytes in vivo [43,44]. Thus, studies mostly focused on the CVPC paracrine function enabling cardiac organ homeostasis [31,45,46,47], for which the injury triggered recruitment would be necessary. Interestingly, it was recently shown that the CVPC transplantation or even just the secreted exosomes are beneficial to the dystrophic heart [48,49]. While the beneficial effect of CVPCs was demonstrated, the dynamics and recruitment of the heart’s own CVPC population remained unclear.

**Figure 7:**
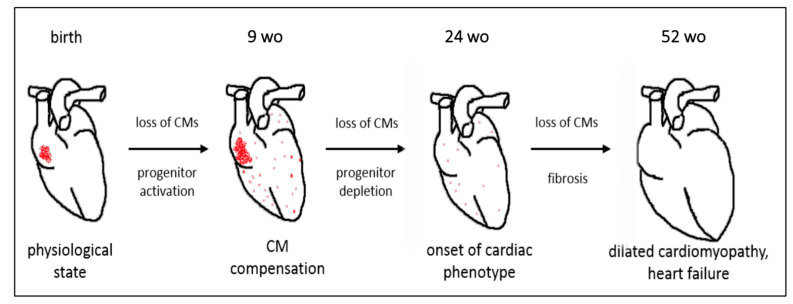
Diagram of the proposed mechanism for the fate of CVPCs in the dystrophin-deficient heart. Dystrophin deficiency induces CVPC proliferation and hyperplasia in 9 wo *mdx* mice associated with an age-dependent depletion and simultaneous progressive fibrosis of the heart during ageing leading to development of cardiac dysfunction by 52 wo.

Our findings demonstrate that dystrophin deficiency induces a CVPC proliferation in young *mdx* mice, consistent with the CVPC proliferation in failing human hearts [50,51]. With ageing, we observed a depletion of CVPC population, consistent with the decrease of the progenitor cells in dystrophin-deficient skeletal muscle [20]. Therefore, although CVPCs and satellite cells have different functions, our findings likely reveal a common phenomenon involving an early-stage recruitment triggered by cardiac injury as also previously shown [34,52], but followed by rapid depletion of the CVPC pool. We summarize our findings and a proposed mechanism of the CVPC in the dystrophin-deficient heart in the diagram of Figure 7.

Some studies showed that myocyte apoptosis precedes the fibrosis in both *mdx* skeletal muscle [53,54] and cardiac muscle as well as in DMD patients [55,56,57,58]. It has been also shown that the cardiomyocyte damage is caused by dystrophin deficiency, chronic inflammatory response, and collagen deposition [13,59,60]. Based on these claims, we hypothesized that increased fibrosis in old *mdx* hearts would impair the enzymatic heart digestion via the Langendorff perfusion. Although we observed increased fibrotic tissue in the old *mdx* hearts, the time for digestion was unchanged. The old WT hearts exhibited less fibrosis but increased digestion time due to some unexpected blood clots not seen in *mdx*. The production of fibrosis in the heart is mainly ensured by the cardiac fibroblasts that are activated in myofibroblasts at the site of injury [61]. It is very likely that the cardiac fibroblast activation causes the progressive increase in fibrosis we observed in *mdx* mice. In parallel, the expression of the matrix metalloproteinases, in particular the MMP-9 (also called gelatinase B), was shown to be upregulated in the dystrophin-deficient heart [62]. However, their activity has been revealed to be dysregulated in dystrophin/utrophin-deficient mice [61]. In the present study, we did not find any difference in the MMP-9 protein expression, although a tendency for higher expression was seen in the old (52 wo) *mdx* heart. Based on these previous studies, we cannot exclude that the MMP-9 dysregulation plays a role in the pathological fibrotic progression and CVPC depletion.

DMD is characterized by the absence of full-length dystrophin (Dp427) in myocytes, including cardiac myocytes. One group revealed retarded maturity and impaired contractile properties in DMD hiPSC-CMs lacking Dp427, suggesting functional consequences at the early stage of cardiac development [63]. Here, we evaluated the dystrophin isoforms expressed in the human and mouse CVPCs and found a key difference in Dp427 expression. The human control CVPCs express several dystrophin isoforms, including Dp427 and ubiquitous isoform Dp71. In contrast, the mouse WT CVPCs only express shorter isoforms including bands at 260, 140, 116, and 71 kDa. The reason of such difference between mouse and human CVPCs is unknown and would require further experiments. Based on several previous studies, including ours, *mdx* mice developed cardiac dysfunction by 20 wo, with myocardial fibrosis, increased collagen expression, ventricular hypertrophy, and arrhythmias as well as left ventricular dysfunction [13,64,65,66,67,68]. These studies clearly support the age-dependent cardiac dysfunction in the *mdx* mice. Here, for the first time, we found that cardiomyocyte damage is associated with increased proliferation of the CVPCs and recruitment into the damaged sites. Although we did not observe any dramatic change in the extracellular matrix component production and inflammation, the evaluation of the age-dependent cardiac dysfunction in *mdx* mice using STE revealed a decrease of LV radial global strain in *mdx* mice at 52 wo, suggesting a cardiac dysfunction. Hence, *mdx* mice are known to have a mild global cardiac phenotype, despite cellular cardiac dysfunctions as early as few weeks of age as we have shown before [13]. Our results show that conventional echocardiography indicates normal cardiac function in *mdx* and WT mice. Using conventional ultrasound measures, cardiac dysfunction usually appears at a late stage after 48 wo in *mdx*. In both groups, a tendency to slow decrease in LVFS was observed from 40 to 30%. To compare, Spurney et al. previously described similar LVFS (32.2%) at 48 wo in WT mice [69], which is concordant with our results. Advanced imaging techniques, such as cardiac magnetic resonance imaging, Doppler tissue imaging or two-dimensional (2D) speckle-tracking echocardiography (STE), and cardiac distortion have shown cardiac dysfunction as early as the age of 32 wo in *mdx* [70,71,72]. Our data confirm these previous studies. STE imaging can be used to quantitatively measure the myocardial strain for potentially sensitive markers for early myocardial dysfunction in *mdx* mice. There is a major difference between conventional fractional shortening determination and the LV segmental STE analysis that takes into account the deformation of all segments. Moreover, the fractional shortening depends on loading conditions and heart rate. The STE analysis is not sensitive to loading conditions or heart rate.

As the functionality of the myocardium decreases, the CVPCs become sparse. These CVPCs possibly contribute to the cardiac muscle homeostasis maintenance via processes such as paracrine signalization by exosome secretion of cardiac cytokines improving damaged myocardium survival as previously proposed [45,46,47,73,74,75,76,77] or even endothelial differentiation and increased vascularization [78] employing progenitor populations as previously shown (e.g., Sca1 and/or c-kit+ cells) [79].

Furthermore, we recently showed that the delayed but progressive cardiac phenotype accelerated in the second decade of life may suggest involvement of CVPC depletion in the heart of patients with Becker muscular dystrophy [37], a milder form of muscular dystrophy than DMD. The first signs of the defective stem cell population in DMD were shown as defective asymmetric division due to dystrophin deficiency limiting the production of the satellite cells in *mdx* skeletal muscles via the Mark2 and EGFR/AurkA kinases [16,80]. In another study, we also showed that deregulated NOS causes damage in the DMD human pluripotent stem cell genome via ROS accumulation [42]. ROS production induces DNA mutations [81] via oxidative DNA damage [82]. This is in agreement with previous findings showing elevated ROS in dystrophic cardiomyocytes and skeletal myocytes [13,83]. We have shown that ROS level plays a role in the satellite cell survival in dystrophin-deficient skeletal muscle [84]. Here, we evaluated the level of oxidative stress in CVPCs but found no significant difference between WT and *mdx* mice. Of note, a similar result was obtained using *mdx* cardiosphere-derived cells that have similar properties to CVPCs. They found no difference either in expression of stem cell markers or myogenic differentiation potential, but both groups were impaired with ageing [85]. The lack of difference could also possibly be caused by the strong antioxidant properties of the supplements added into the culturing medium of the CVPCs, such as vitamins and selenium, which are essential components of several antioxidant enzymes [86,87,88]. As other groups have revealed the presence of oxidative stress in the *mdx* cardiomyocytes and its role in the related DCM development [64,89], we cannot exclude the possibility that the *mdx* CVPCs exhibit oxidative stress that contributes to increased DNA damage at 24 wo, right before the CVPC population depletion.

We thus suggest that impaired cardiac function in *mdx* mice, as we have shown here and previously [13], and shorter cardiomyocyte lifespan [54] combined with inflammation [59] and collagen deposition lead to upregulation of CVPC proliferation in young *mdx* mice. At the same time, normal CVPCs express dystrophin similarly to the muscle satellite cells [16,90] and human pluripotent stem cells [42]. Dystrophin deficiency may thus weaken CVPCs as demonstrated by accumulation of DNA damage, and eventually cause their depletion in the heart. Thus, the dystrophin deficiency not only impairs the CVPC pool but also causes DNA damage in this pool. Altogether, there are fewer CVPCs in *mdx* mice, and these cells are damaged with age. Such a mechanism was described in depletion of other tissue-specific stem cells, including melanocyte and hematopoietic stem cells [91,92,93]. Although no information on CVPC mutagenesis is available, the DNA damage may lead to accelerated ageing [94,95], subsequent depletion, and diminished stem cell potential, including paracrine signaling and homeostatic function of the CVPC niche. Such a mechanism is in concert with lower CVPC expansion and differentiation capacity, shortened lifespan, and premature senescence in the DMD (GRMD) canine model [15]. While at early stage the CVPCs may have a paracrine function for cardiac regeneration, their disappearance may then lead to rapid deterioration, finally exacerbated by fast progressing cardiac fibrosis.

## 4. Materials and Methods

### 4.1. Ethical Statements

Animal procedures were conducted according to the guidelines of the Declaration of Helsinki, approved by the Institutional Ethics Committee for animal experiments, and received agreement from the national “Ministère de l’ Enseignement Supérieur et de la Recherche” (No. #16473-2018082016141320). This research complied with the commonly accepted ”3Rs”. C57BL/10-Dmd*^mdx^* male mice were chosen because of the appropriate lifespan to follow up the CVPC pool in the dystrophin-deficient heart. They were obtained from a colony maintained at a local animal facility network (RAM, Montpellier) [96]. C57BL/6 mice were purchased from Janvier Laboratories (Le Genest-Saint-Isle, France). Animals were housed conventionally in controlled humidity and temperature, operating on 12 h of light and dark cycles with food and water available ad libitum. Overall, 76 WT and 74 *mdx* mice were used for this study.

### 4.2. Immunohistology

The mice were sacrificed by cervical dislocation, and the heart was extracted from the chest. The organ was placed onto a Langendorff setup for aorta cannulation and perfused at 37 °C with a modified Tyrode solution (113 mM NaCl, 4.7 mM KCl, 0.6 mM KH_2_PO_4_, 0.6 mM Na_2_HPO_4_, 1.2 mM MgSO_4_·7H_2_O, 12 mM NaHCO_3_, 10 mM KHCO_3_, 10 mM HEPES, 30 mM Taurine) with heparin (50 µL of 1000 U/mL solution) to remove blood residue. The heart was then removed and flash frozen in isopentane cooled by liquid nitrogen for cryoprotection of the tissues. The hearts were sectioned (5 μm) on a cryotome at a temperature of −20 to −25 °C. At least 20 (and up to 100) sections were analyzed for each animal. For histological staining of collagen deposits, a Masson’s trichrome staining protocol was used. Briefly, sections were fixed with 4% paraformaldehyde and stained with Wiegert’s hematoxylin. Then, the samples were fixed in Bouin’s solution for 1 h and stained with fuchsin, differentiated in phosphomolybdic/phosphotungstic acid, and collagen was stained with aniline blue. Samples were then dried, clarified with xylene, and mounted with Pertex. The images were taken with an automatic TissueFAXS system (Tissuegnostics, Vienna, Austria) and Observer.Z1 microscope (Carl Zeiss, Oberkochen, Germany), and images were analyzed using QuPath software [97]. For the analysis of CVPC presence, the sections were fixed with 4% paraformaldehyde, incubated in blocking solution (0.1% BSA, 1% goat serum, 0.2% triton-X in 1× PBS), and then with primary antibodies against CD117 (AF1356, 1:100, R&D) and CD45 (cl. 30-F11, NB-100-77417SS, 1:50, Novus Biologicals, Cenntenial, CO, USA) overnight. Secondary antibodies (anti-rat Alexa488, anti-mouse Alexa594, Invitrogen, both 1:500) were applied after washing for one hour at room temperature. Nuclei were counterstained with DAPI. The microscopic images were taken with the automatic Tissue FAXS system (Tissuegnostics) and Observer.Z1 microscope (Carl Zeiss) for the whole overview and focused areas. Detailed images of areas of interest were acquired with an LSM700 confocal microscope system (Carl Zeiss).

### 4.3. Mouse Heart Dissociation

Heart dissociation was conducted according to the Langendorff method [98] with buffer containing 0.1 g/mL liberase/dispase (Roche, France) as previously described [13]. Isolated cells were transferred to an enzyme-free solution containing 1 mM CaCl_2_ and 10% FBS, and the small cell population was separated from the myocytes with a cell strainer of 40 µm (Corning, New York, NY, USA). 

### 4.4. CVPC Analysis

Following mouse heart dissociation, the suspension of small cells was labeled with CD45 (130-052-301) and CD117 (130-091-224) microbeads to obtain the desired population of CD117^+^/CD45^−^ cells with MACS columns (Miltenyi Biotec, Bergisch Gladbach, Germany). These cells were resuspended in CVPC medium (90% DMEM/F12 with glutamine (D8437, Sigma-Aldrich, Saint Louise, MO, USA), 10% ESC FBS (ES-009-B, Merck Millipore, Burlington, MA, USA), 1% penicillin/streptomycin, 1% insulin/transferrin/selenium, 10 ng/mL mouse bFGF, 10 ng/mL mouse EGF, 50 μg/mL gentamycin) and left to attach onto gelatin coated cover slips. The cells were analyzed within 10 days after isolation. For control of protein expression, a human sample of CVPCs was used. Following informed consent of the patient, discarded human right atrial appendages were collected during routine open heart procedures from Montpellier University Hospital, Arnaud de Villeneuve. Collection of the tissues followed the protocol approved by the French Ministry of National Education and Research (DC-2013-2023). CVPCs were isolated following the protocol in [99]. Fat was removed from atrial appendage specimens, and after washing with cold PBS, the tissues were cut into small pieces (roughly 1 mm). The minced tissue was enzymaticallly digested with collagenase II (30 U/mL; Thermo Fisher, Waltham, MA, USA) in PBS at 37 °C, shaking for one hour after which the remaining tissue was mechanically digested. The undigested tissue was left to settle by gravity, and the supernatant containing the CVPCs was collected. A second round of enzymatic and mechanical digestion was performed on the remaining tissue fragments. CVPCs were pelleted, resuspended, and plated in growth media: Ham’s F12 (Sigma-Aldrich), 10% FBS (Hyclone, Logan, UT, USA), 10 ng/mL bFGF (Miltenyi Biotec), 0.2 mM glutathione (Sigma-Aldrich), 0.005 U/mL erythropoietin (Sigma), and penicillin/streptomycin. At passage 2, CVPCs were magnetically sorted using the Miltenyi Biotec MACS magnet for the CD117^+^ population.

### 4.5. ROS and DNA Damage Analysis

For the detection of ROS, the adherent cells were incubated with CellROX Green reagent (Life technologies, detecting both ROS and RNS) for 1 h. Cells were then harvested using 0.5 mM EDTA solution and counted. Fluorescence in the green spectrum was measured using the monochromometer Tecan Infinite 200 PRO, and the signal of green was normalized by the total number of cells.

For immunofluorescent labeling of proteins, cells were cultivated on glass cover slips covered in gelatin, washed with PBS, and fixed using 4% paraformaldehyde at room temperature for 15 min. Cells were incubated with pre-extraction buffer (25 mM HEPES, 50 mM NaCl, 1 mM EDTA, 3 mM MgCl2, 200 mM sucrose, 0.5% Triton X-100) before continuing to blocking steps. The cells were permeabilized and blocked with blocking solution 1 (1% BSA, Triton X-100 0.2% in PBS, 15 min on ice) and blocking solution 2 (1% BSA, 1% NaN3, 0.05% Tween 20 in PBS, 60 min on ice). γH2AX Ser139 antibody (mouse monoclonal, 613402, BioLegend, San Diego, CA, USA) was incubated overnight at 4 °C. Samples were extensively washed with PBS/Tween (0.05%) and incubated with a fluorescently labeled secondary antibody against mouse for 1 h at room temperature. DAPI was used as a nuclear counterstain. Microscopy was performed using a confocal LSM700 microscope (Carl Zeiss), and images were processed using the ZEN system (Carl Zeiss). For analysis of DNA double stranded breaks, the total number of γH2AX foci per image was divided by the total nuclei count per image.

### 4.6. Quantitative RT-PCR

For total c-kit expression in the heart, quantitative PCR was performed on frozen left ventricle samples of the chosen age WT and *mdx* groups. Total mRNA was extracted using the NucleoSpin RNA kit (Macherey-Nagel, Hoerdt, France) followed by reverse transcription with the RevertAid RT Reverse Transcription Kit (Thermo Fisher). qPCR was performed using LightCycler^®^ 480 SybrGreen I Master (Roche) and a LightCycler^®^ 480 Instrument II (Roche Life sciences). We used the following primers: c-kit forward 5′-ACTCGCACGGGGACATA-3′and reverse 5′-GGAGAGATTTCCCATCACACT-3′, the ribosomal protein large subunit P0 (*Rplp0*) as the housekeeping gene, forward 5′-ACATCATCCCTGCCTCTAC-3′, and reverse 5′-CCTGCTTCACCACCTTCTT-3′. The presented value was calculated as ΔΔCt compared to housekeeping gene and 9 wo WT animals.

### 4.7. Western Blot Analysis

Protein extracts in radioimmunoprecipitation assay (RIPA) buffer were separated by SDS-PAGE 8–10% gradient gel electrophoresis and transferred overnight at 4 °C to nitrocellulose membranes, blocked at room temperature with Odyssey blocking buffer (Eurobio, France) and probed with a H4 (1/1000) rabbit polyclonal anti-dystrophin [100], rabbit polyclonal anti-histone H1.4 (Sigma-Aldrich; 1/5000, ref H7665), and mouse monoclonal anti α-tubulin (Sigma-Aldrich, 1/1000, ref: T9026) followed by IRDye^®^ 680RD (α-tubulin) or IRDye^®^ 800RD (H1.4) secondary antibodies (Eurobio, Les Ulis, France). Fluorescence was quantified with the Odyssey software (West Henrietta, NY, USA). Data were normalized to histone H1.4 expression and α-tubulin. For collagen and inflammation markers, cardiac tissue was homogenized in RIPA buffer and separated by SDS-PAGE 10% gel electrophoresis and transferred to nitrocellulose membranes. After blocking at room temperature with 5% milk, membranes were incubated with primary antibodies against collagen 1A1 (COL1A1, rabbit polyclonal, ref PA5-29569, Thermo Fisher), collagen 3 (rabbit polyclonal, ref PA5-34787, Thermo Fisher), mouse matrix metalloproteinase 9 (MMP-9, goat polyclonal, ref AF909-SP, R&D), and human/Mouse cyclooxygenase 2 (COX-2, goat polyclonal ref AF4198-SP, R&D). The GAPDH antibody (rabbit polyclonal, ref 2118, Cell Signal) was used as loading control for these markers.

### 4.8. Echocardiography

Cardiac function was evaluated in vivo in *mdx* and WT (C57BL/10ScSnJ) mice from 8 to 48 week-old (wo) (*n* = 10) by 2D transthoracic echocardiography using a high frequency ultrasound system (Vevo 2100, Visualsonics, Toronto, ON, Canada) equipped with a probe (30 MHz) operating at a frame rate of 200–250 per second. M-mode images were obtained using the LV short-axis view at the mid papillary muscle level. The M-mode gate was set in the middle between the two papillary muscles for fractional shortening analysis. For 2D STE studies, the short-axis B mode at the mid LV level and long-axis B mode cine loops were recorded with a scanhead placed at the mid-LV level, i.e., along the left sternal border with a clear view of both the apex and the outflow tract of LV. Three consecutive 2D videos of cardiac and stable cycles were recorded. Recordings were performed in anesthetized mice (1.5% isoflurane, 100% O_2_). Animals were placed in a dorsal position on a heat-warming tray (37 °C) controlling heart rate (HR) > 400 beats/min for the duration of the study (with adjustment of level of anesthesia as necessary). All measurements and calculations were performed with the Vevo 2100 Software and were averaged from a minimum of three cycles and followed the standards of the American Society of Echocardiography and the Vevo 2100 guidelines.

### 4.9. Statistical Analysis

Normality was tested using the Shapiro–Wilk test. An unpaired *t*-test was used to compare 2 independent groups with parametric distribution. Two-way ANOVA was performed to analyze the age and mutation involvement in analyzed groups. Specific tests for each analysis are reported in individual figure legends. All data are expressed as mean ± SEM. A value of *p* < 0.05 was considered significant. Prism 6 (GraphPad, San Diego, CA, USA) was used for data analysis and statistics.

## 5. Conclusions

Dystrophin deficiency induces changes in *mdx* heart leading to cardiac dysfunction and excessive recruitment of CVPCs in injured sites, likely to maintain the tissue homeostasis. The dystrophinopathy impairs the CVPC pool by increasing DNA damage and need for extensive proliferation, causing early exhaustion of the cardiac tissue homeostasis maintenance capacity. Such a mechanism of progressive CVPC pool depletion, together with the cardiac pathophysiological condition associated with DMD, may be the major reason for progressive fibrosis in the heart of the DMD patients.

## 6. Contribution to the Field

Early cardiac dysfunction in DMD is clinically associated with left ventricular strain reduction due to fibrosis, hypocontractility, and arrhythmias. This leads to fully developed cardiomyopathy, which has become the leading cause of mortality in DMD patients. The cardiac dysfunction is primarily attributed to cardiomyocyte damage. This mechanism, however, is not sufficient to fully explain the delayed onset of DCM in the teenage years. Our present results reveal a link between CVPC activation in the heart and the progressive damage upon their depletion. The CVPCs are thought to primarily differentiate into an endothelial lineage but can provide important positive paracrine effects to the damaged myocardium. Our findings reveal a possible role of the CVPCs in the age-dependent cardiac dysfunction, which may eventually provide a target to improve the cardiac muscle homeostasis maintenance and lifespan in DMD patients. Our findings may help to reveal the molecular mechanisms involving the CVPCs that might be shared in other cardiac disorders.
